# Triglyceride-glucose index and cervical vascular function: outpatient-based cohort study

**DOI:** 10.1186/s12902-023-01449-5

**Published:** 2023-09-08

**Authors:** Xiaoyu Pan, Lin Yue, Lin Ren, Jiangli Ban, Shuchun Chen

**Affiliations:** 1 Department of Internal Medicine, Hebei Medical University, Shijiazhuang, Hebei China; 2 Department of Endocrinology, Hebei General Hospital, Shijiazhuang, Hebei China; 3 Department of Endocrinology, The Third Hospital of Shijiazhuang, Shijiazhuang, Hebei China

**Keywords:** Triglyceride-Glucose Index, Internal Carotid Artery, Basilar Artery, Vertebral Artery, Insulin Resistance

## Abstract

**Objectives:**

The purpose of this study was to investigate the correlation between triglyceride-glucose (TyG) index and cervical vascular function parameters in the general population without cerebrovascular disease.

**Materials and Methods:**

This was a cross-sectional study that recruited a total of 1996 participants without cerebrovascular disease. TyG index was calculated based on fasting triglycerides and glucose. All patients were divided into two groups based on the median TyG index: the high TyG group and the low TyG group. The differences in basic clinical characteristics and neck vascular function parameters between the two groups of participants were compared, and then the correlation between TyG index and neck vascular function parameters was investigated.

**Results:**

Participants with a high TyG index had lower systolic, diastolic, and mean flow velocities in the basilar, vertebral, and internal carotid arteries compared with those with a low TyG index. Participants with a high TyG index had higher pulsatility index in the left vertebral artery and right internal carotid artery, but this difference was not observed in the basilar artery. In addition, TyG index was significantly negatively correlated with systolic, diastolic, and mean flow velocities in the basilar, vertebral, and internal carotid arteries, and the correlation remained after adjusting for confounding factors.

**Conclusion:**

In the general population, there was a well-defined correlation between TyG index and cervical vascular function parameters, and increased TyG index was independently associated with reduced cervical vascular blood flow velocity.

## Introduction

Cerebrovascular disease is among the principal ailments that have an impact on the health and quality of life of middle-aged and older people [1, 2]. The phrase "cerebrovascular disease" refers to a category of conditions where cerebrovascular lesions result in localized or widespread brain dysfunction. The most frequent cerebrovascular lesions are those that produce either diffuse or restricted brain dysfunction, such as blockage or narrowing of the vascular lumen, damage to the vascular wall, or changes in permeability. Early cerebrovascular function screening can thereby significantly improve patient quality of life, significantly save healthcare costs, and reduce the risk of acquiring cerebrovascular disease. Transcranial Doppler (TCD) ultrasound, which is easy to use, repeatable, and capable of providing continuous, long-term dynamic surveillance of the patient, overcomes the inaccuracy of cerebral hemograms and the invasiveness of cerebral angiography during routine physical examinations. It provides reference data for the diagnosis, monitoring, and treatment of cerebrovascular disease and is essential in determining the functionality of the basilar artery (BA), carotid artery (CA), and vertebral artery (VA) [3–5]. Also, earlier research has demonstrated the value of the vascular parameters picked up by TCD as diagnostic and therapeutic tools for clinical cerebrovascular disorders [6, 7].

In order to maintain stable blood glucose levels, the body must create excessive amounts of insulin, which leads to hyperinsulinemia. This state is known as insulin resistance (IR). When insulin's ability to boost glucose uptake and utilization begins to decline for a variety of reasons, IR happens. The "gold standard" for the diagnosis of IR is the hyperinsulinemic-euglycemic clamp, which assesses glucose disposal rate. Nevertheless, because to the complexity of the method, it is rarely used in clinical practice [8]. The triglyceride-glucose (TyG) index is created using the ln(fasting triglyceride[mg/dL]*fasting glucose[mg/dL]/2) formula, which is currently believed to be a more accurate indicator of IR [9]. Previous studies [10–13] have shown a connection between the TyG index and nonalcoholic fatty liver, diabetic nephropathy, and cardiovascular disease. Animal and clinical observational investigations have demonstrated that IR is closely linked to functional abnormalities in CA, VA, and BA [14–16]. IR affects a range of vascular processes. IR causes enhanced SGK-1 signaling to worsen vascular stiffness and inhibits protein kinase C to evoke vascular wall inflammation and atherosclerosis progression [17, 18]. In addition, IR may lead to an imbalance between NO production and endothelin-1 secretion, resulting in endothelial dysfunction, which can be restored by improving IR [19]. Several clinical studies have demonstrated a high correlation between the TyG index and stroke, as well as a correlation with carotid intima-media thickness [20–22].

In the general population without cerebrovascular disease, the relationship between TyG index and vascular function as determined by TCD is uncertain. The goal of this study was to investigate the relationship between the TyG index and functional measures of the BA, internal carotid artery (ICA), and VA in the general population. This work might offer fresh perspectives on how to identify, diagnose, and treat cerebrovascular disorders in the general population early on.

## Materials and methods

### Study population

This is a cross-sectional study. The general population without cerebrovascular disease who underwent physical examinations at the Hebei Provincial Health Examination Center from June to July 2022 were recruited. An informed consent form was signed by each participants. Screening of the study population was performed independently by two investigators. The study was conducted in accordance with the Declaration of Helsinki and was approved by Hebei General Hospital Ethics Committee (Ethics Committee No. 202285).

All general population who underwent physical examination were considered as potential study subjects. Exclusion criteria: (1) Age ≤ 18 years old; (2) participants with severe liver and kidney dysfunction, malignant tumors; (3) Pregnant or breastfeeding female participants; (4) Pre-existing history of cerebrovascular disease; (5) History of cervical vascular surgery; (6) Drug use, such as antiplatelet drugs, antihypertensives, vasodilators, and other drugs that affect vascular function.

### Information and biomarkers collection

Baseline clinical information, including age and gender, was collected from all participants. Height, weight, systolic blood pressure (SBP) and diastolic blood pressure (DBP) (yuwell sphygmomanometer, YE670AR) were measured three times and the mean values were recorded. Blood samples from participants fasting for 8 h were collected to test for blood indicators. Total cholesterol (TC), triglycerides (TG), low-density lipoprotein cholesterol (LDL-C), high-density lipoprotein cholesterol (HDL-C), fasting blood glucose (FBG), and glycosylated hemoglobin (HbA1c) were measured in the same laboratory (Beckman Coulter AU5800 Automated Biochemistry Analyzer).

### Calculation of parameters

The TyG index is calculated from TG and FBG levels by the formula Ln (fasting TG (mg/dl) × fasting blood glucose (mg/dl)/2). Body mass index (BMI) = weight (kg)/height (m)^2^.

### Transcranial Doppler Ultrasound

Transcranial Doppler Ultrasound directly traces the Doppler signal of the blood flow in the fundic arteries with the aid of pulsed Doppler technology and a 2 MHz emission frequency. The flow velocities of the internal carotid, basilar and vertebral arteries as well as the pulsatility index are measured. All indices were recorded according to the same criteria and the average of 3 measurements was taken as the final value.

### Statistical analysis

All data were analyzed and visualized using GraphPad Prism 8.01 software and SPSS 26 software. All data were tested for normality and expressed as mean ± standard deviation if they satisfied the normal distribution and vice versa as median (25th percentile, 75th percentile). Differences between groups were tested using Student's t-test. If the data did not conform to a normal distribution use Mann–Whitney U analysis for comparison of differences between groups. Spearman or Pearson correlation analysis to determine the correlation between variables. Multiple linear regression explores independent correlations between variables. When P < 0.05 considered the difference between groups was statistically significant.

## Results

### Clinical Characteristics of All Participants

A total of 1996 (1248 male) participants were included in the study. The median age was 58 years, BMI was 24.54 kg/m^2^, and FBG was 5.625 mmol/L. The median TyG index was 8.71, and based on this value all participants were divided into a high TyG group and a low TyG group, with 998 participants in each group. The basic clinical characteristics and cervical vascular parameters of all participants are shown in Table 1.

**Table 1:** Clinical characteristics of all participants

	Subjects (*n* = 1996)
Gender (Male)	(1248, 62.53%)
Age (y)	58 (49, 69)
BMI (kg/m^2^)	24.54 (22.6, 26.67)
SBP (mmHg)	125 (113, 137)
DBP(mmHg)	78 (71, 86)
TC (mmol/L)	4.895 (4.22, 5.548)
TG (mmol/L)	1.32 (0.95, 1.85)
LDL-C (mmol/L)	3.08 (2.59, 3.578)
HDL-C (mmol/L)	1.29 (1.11, 1.498)
FBG (mmol/L)	5.625 (5.23, 6.22)
HbA1c (%)	5.8 (5.6, 6.2)
TyG index	8.71 (8.362, 9.111)
BA pulsatility index	0.86 (0.75, 0.99)
BA systolic blood flow	56 (46, 67)
BA diastolic blood flow	24 (19, 30)
Mean BA blood flow	35 (28, 42)
Right ICA pulsatility index in proximal cranial segment	0.9 (0.79, 1.04)
Right ICA systolic flow velocity in proximal cranial segment	58 (51, 67)
Right ICA diastolic flow velocity in proximal cranial segment	25 (21, 29)
Mean right ICA flow velocity in proximal cranial segment	36 (31, 41)
Right VA pulsatility index in intracranial segment	0.85 (0.75, 0.97)
Right VA systolic flow velocity in intracranial segment	46 (41, 54)
Right VA diastolic flow velocity in intracranial segment	21 (18, 24)
Mean right VA flow velocity in intracranial segment	29 (26, 34)
Left ICA pulsatility index in proximal cranial segment	0.89 (0.78, 1.03)
Left ICA systolic flow velocity in proximal cranial segment	59 (52, 68)
Left ICA diastolic flow velocity in proximal cranial segment	25 (21, 30)
Mean left ICA flow velocity in proximal cranial segment	37 (32, 42)
Left VA pulsatility index in intracranial segment	0.83 (0.73, 0.95)
Left VA systolic flow velocity in intracranial segment	47 (41, 54)
Left VA diastolic flow velocity in intracranial segment	21 (18, 25)
Mean left VA flow velocity in intracranial segment	30 (26, 34)

*BMI* body mass index, *SBP* systolic blood pressure, *DBP* diastolic blood pressure, *TC* total cholesterol, *TG* triglyceride, *LDL-C*, low-density lipoprotein cholesterol, *HDL-C*, high-density lipoprotein cholesterol, *FBG* fasting blood glucose, *HbA1c* glycated hemoglobin, *TyG index* triglyceride-glucose inde, *BA* Basilar artery, *ICA* internal carotid artery, *VA* vertebral artery

### Comparison of clinical characteristics in the low TyG and high TyG groups

**Fig. 1:**
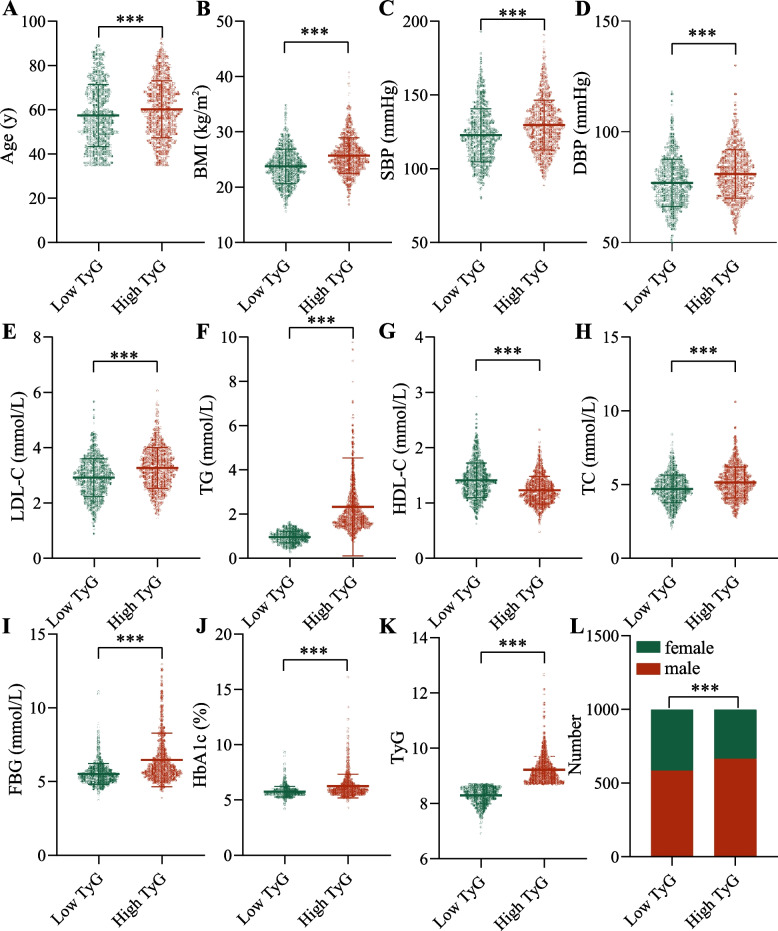
Comparison of baseline characteristics between the high TyG and low TyG groups. (**A**) Age. (**B**) BMI. (**C**) SBP. (**D**) DBP. (**E**) LDL-C. (**F**) TG. (**G**) HDL-C. (**H**) TC. (**I**) FBG. (**J**) HbA1c. (**K**) TyG. (**L**) Histogram of the number of males and females. Abbreviations: TyG, triglyceride-glucose; BMI, body mass index; SBP: systolic blood pressure; DBP: diastolic blood pressure; LDL-C: low-density lipoprotein cholesterol; TG: triglyceride; HDL-C: high-density lipoprotein cholesterol; TC: total cholesterol; FBG: fasting blood glucose; HbA1c, glycosylated hemoglobin. ***P < 0.001

Participants in the high TyG index group had greater age and BMI (Fig. 1 A and B), as well as having higher SBP and DBP compared to participants in the low TyG index group (all *P* < 0.001) (Fig. 1 C and D). For lipids, participants with high TyG index had higher TC, TG and LDL-C and lower HDL-C compared to those with low TyG (all *P* < 0.001) (Fig. 1 E–H). FBG and HbA1c were higher in the high TyG group (all *P* < 0.001) (Fig. 1I and J). Since the grouping was based on the median TyG, the high TyG group had a significantly higher TyG index than the low TyG group, and the high TyG group had a higher proportion of males (all *P* < 0.001) (Fig. 1 K and L).

### BA functional parameters in high TyG and low TyG groups

**Fig. 2:**
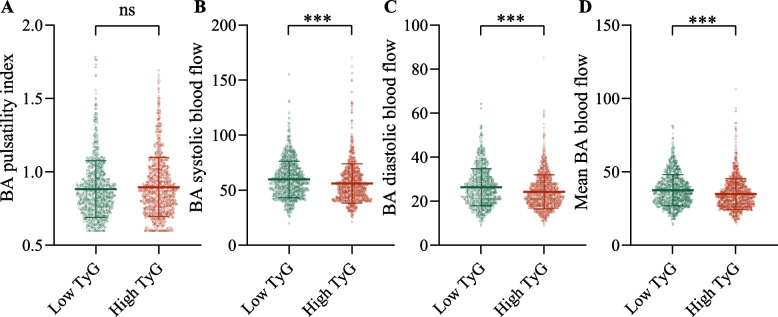
Comparison of BA functional parameters between the high TyG and low TyG groups. (**A**) BA pulsatility index. (**B**) BA systolic blood flow. (**C**) BA diastolic blood flow. (**D**) Mean BA blood flow. Abbreviations: TyG, triglyceride-glucose; BA, Basilar artery. ns, *P* > 0.05. ***P < 0.001

There seems to be no significant effect of TyG index on BA pulsatility index (*P* > 0.05) (Fig. 2A). Participants in the high TyG group had significantly lower BA systolic, diastolic and mean blood flow velocities compared to participants in the low TyG group (all *P* < 0.001) (Fig. 2 B-D).

### ICA function parameters in high TyG and low TyG groups

**Fig. 3:**
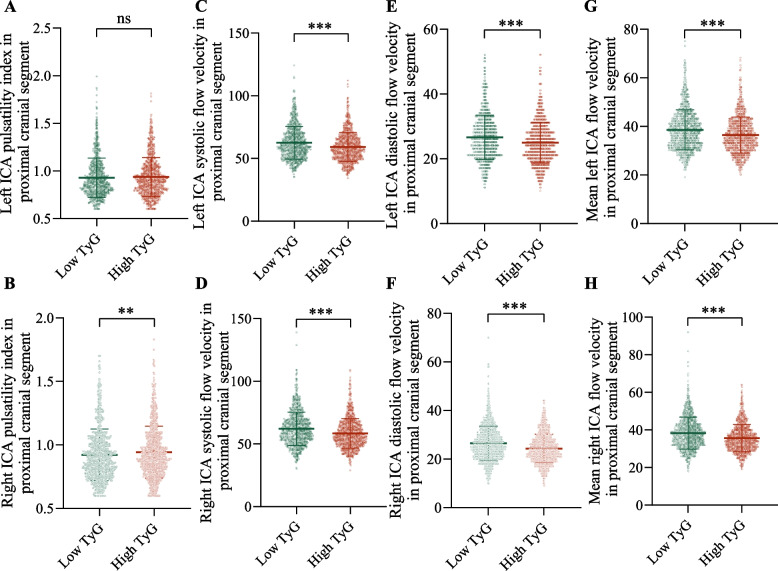
Comparison of ICA functional parameters between the high TyG and low TyG groups. (**A**) Left ICA pulsatility index in proximal cranial segment. (**B**) Left ICA systolic flow velocity in proximal cranial segment. (**C**) Left ICA diastolic flow velocity in proximal cranial segment. (**D**) Mean left ICA flow velocity in proximal cranial segment. (**E**) Right ICA pulsatility index in proximal cranial segment. (**F**) Right ICA systolic flow velocity in proximal cranial segment. (**G**) Right ICA diastolic flow velocity in proximal cranial segment. (**H**) Mean right ICA flow velocity in proximal cranial segment. Abbreviations: TyG, triglyceride-glucose; ICA, internal carotid artery. ns, *P* > 0.05. ***P < 0.001. **P < 0.01

A slightly different effect of TyG index on the ICA pulsatility index was observed on the left and right sides. On the left side, TyG index does not seem to affect the ICA pulsatility index (*P* > 0.05), while on the right side, participants with higher TyG index have higher ICA pulsatility index (*P* < 0.01) (Fig. 3 A and B). Participants with high TyG index had lower ICA systolic, diastolic and mean blood flow velocities compared to those with low TyG regardless of right or left side (all *P* < 0.001) (Fig. 3 C-H).

### VA function parameters in high TyG and low TyG groups

**Fig. 4:**
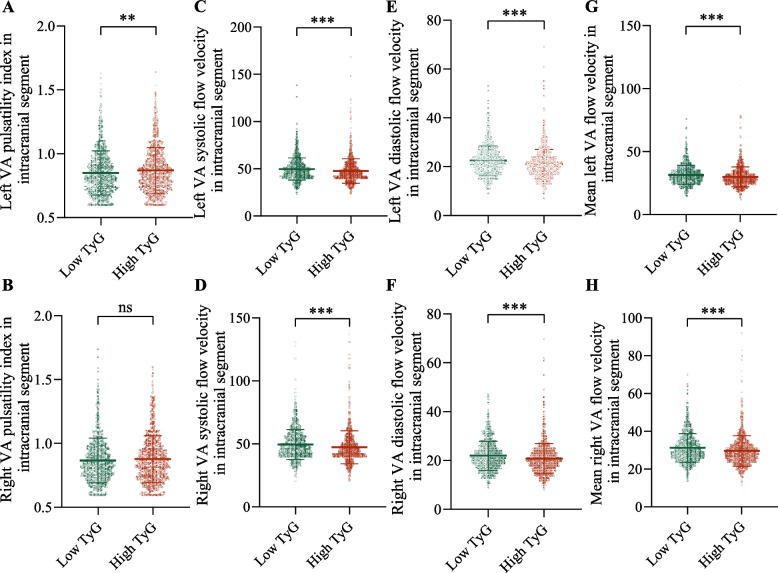
Comparison of VA functional parameters between the high TyG and low TyG groups. (**A**) Left VA pulsatility index in intracranial segment. (**B**) Left VA systolic flow velocity in intracranial segment. (**C**) Left VA diastolic flow velocity in intracranial segment. (**D**) Mean left VA flow velocity in intracranial segment. (**E**) Right VA pulsatility index in intracranial segment. (**F**) Right VA systolic flow velocity in intracranial segment. (**G**) Right VA diastolic flow velocity in intracranial segment. (**H**) Mean right VA flow velocity in intracranial segment. Abbreviations: TyG, triglyceride-glucose; VA, vertebral artery. ns, *P* > 0.05. ***P < 0.001. **P < 0.01

The effect of TyG index on VA pulsatility index was opposite to that of ICA, meaning that participants with higher TyG index had higher VA pulsatility index on the left side (*P* < 0.01) instead of on the right side (*P* > 0.05) (Fig. 4 A and B). High TyG index participants had lower VA systolic, diastolic and mean blood flow velocities regardless of right or left side compared to those with low TyG (all *P* < 0.001) (Fig. 4 C-H).

### Correlation of TyG index with BA, ICA and VA function parameters

**Fig. 5:**
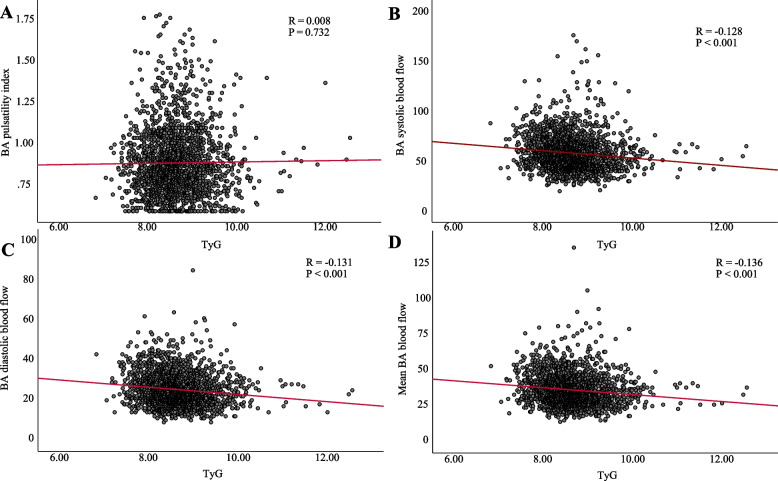
Correlation between TyG index and BA functional parameters. (**A**) BA pulsatility index. (**B**) BA systolic blood flow. (**C**) BA diastolic blood flow. (**D**) Mean BA blood flow. Abbreviations: TyG, triglyceride-glucose; BA, Basilar artery

**Fig. 6:**
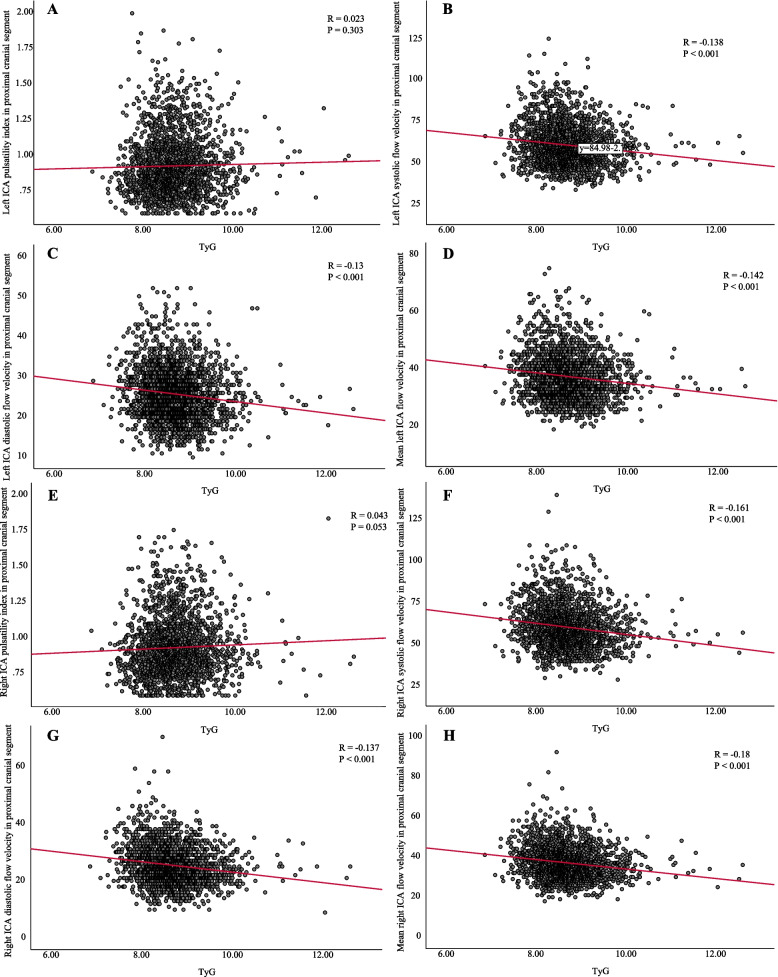
Correlation between TyG index and ICA functional parameters. (**A**) Left ICA pulsatility index in proximal cranial segment. (**B**) Left ICA systolic flow velocity in proximal cranial segment. (**C**) Left ICA diastolic flow velocity in proximal cranial segment. (**D**) Mean left ICA flow velocity in proximal cranial segment. (**E**) Right ICA pulsatility index in proximal cranial segment. (**F**) Right ICA systolic flow velocity in proximal cranial segment. (**G**) Right ICA diastolic flow velocity in proximal cranial segment. (**H**) Mean right ICA flow velocity in proximal cranial segment. Abbreviations: TyG, triglyceride-glucose; ICA, internal carotid artery

**Fig. 7:**
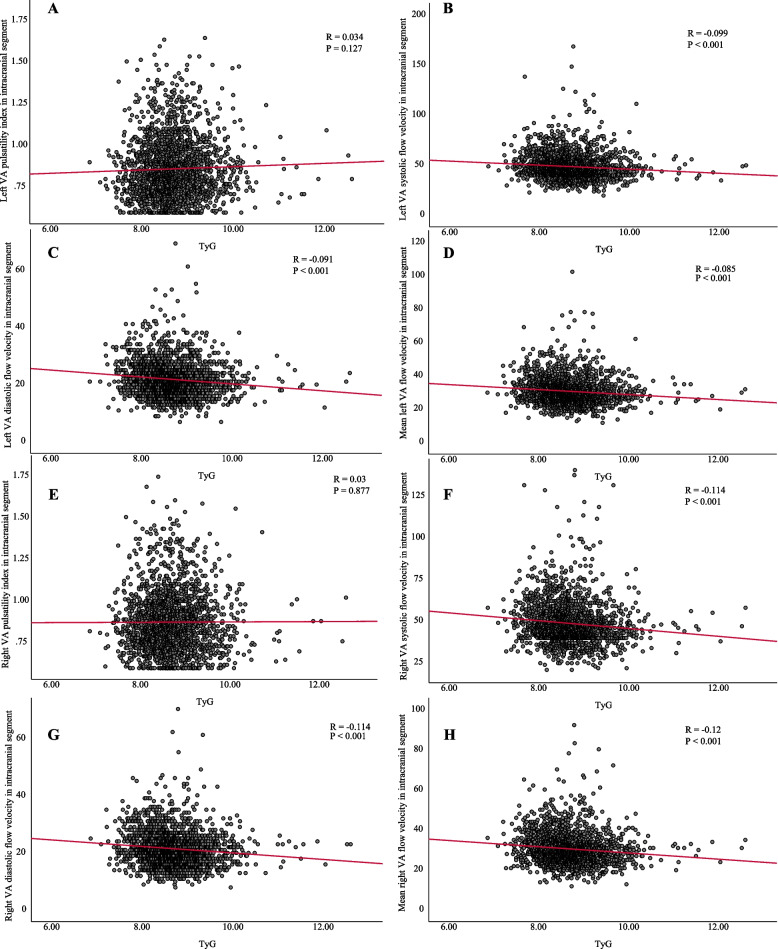
Correlation between TyG index and VA functional parameters. (**A**) Left VA pulsatility index in intracranial segment. (**B**) Left VA systolic flow velocity in intracranial segment. (**C**) Left VA diastolic flow velocity in intracranial segment. (**D**) Mean left VA flow velocity in intracranial segment. (**E**) Right VA pulsatility index in intracranial segment. (**F**) Right VA systolic flow velocity in intracranial segment. (**G**) Right VA diastolic flow velocity in intracranial segment. (**H**) Mean right VA flow velocity in intracranial segment. Abbreviations: TyG, triglyceride-glucose; VA, vertebral artery

Correlation analysis showed a significant negative correlation between TyG index with BA, VA and ICA systolic, diastolic and mean flow velocities (all *P* < 0.001), while no significant correlation was observed between TyG index with BA, VA and ICA pulsatility index (all *P* > 0.05) (Figs. 5, 6 and 7).

### Multivariate linear correlation analysis between TyG index and BA, ICA and VA function parameters

TyG index was negatively correlated with BA, VA and ICA systolic, diastolic and mean flow velocities in crude model 1, model 2 (adjusted for age) and model 3 (adjusted for age, SBP and DBP) (Table 2). BA and right VA pulse indices were negatively correlated with TyG indices in model 2, while not observed in model 1 and model 3. There was no correlation present between left-sided VA and bilateral ICA and TyG index, with or without adjustment for confounders.

**Table 2:** Correlation between TyG index and cervical vascular function after adjustment for confounding factors

	Model 1		Model 2		Model 3	
	t	*p*	t	*p*	t	*p*
BA pulsatility index	0.55	0.582	-2.209	0.027	-1.295	0.195
BA systolic blood flow	-5.774	< 0.001	-5.652	< 0.001	-4.739	< 0.001
BA diastolic blood flow	-6.111	< 0.001	-4.969	< 0.001	-4.219	< 0.001
Mean BA blood flow	-6.118	< 0.001	-5.405	< 0.001	-4.641	< 0.001
Right ICA pulsatility index in proximal cranial segment	1.939	0.053	-0.622	0.534	0.2	0.841
Right ICA systolic flow velocity in proximal cranial segment	-7.629	< 0.001	-7.131	< 0.001	-6.114	< 0.001
Right ICA diastolic flow velocity in proximal cranial segment	-7.615	< 0.001	-6.457	< 0.001	-5.856	< 0.001
Mean right ICA flow velocity in proximal cranial segment	-8.17	< 0.001	-7.333	< 0.001	-6.437	< 0.001
Right VA pulsatility index in intracranial segment	0.156	0.876	-1.974	0.049	-1.46	0.145
Right VA systolic flow velocity in intracranial segment	-5.117	< 0.001	-4.996	< 0.001	-4.325	< 0.001
Right VA diastolic flow velocity in intracranial segment	-5.105	< 0.001	-4.065	< 0.001	-3.607	< 0.001
Mean right VA flow velocity in intracranial segment	-5.402	< 0.001	-4.759	< 0.001	-4.148	< 0.001
Left ICA pulsatility index in proximal cranial segment	1.031	0.303	-1.844	0.065	-0.67	0.503
Left ICA systolic flow velocity in proximal cranial segment	-6.203	< 0.001	-6.093	< 0.001	-4.929	< 0.001
Left ICA diastolic flow velocity in proximal cranial segment	-5.871	< 0.001	-4.496	< 0.001	-4.066	< 0.001
Mean left ICA flow velocity in proximal cranial segment	-6.414	< 0.001	-5.517	< 0.001	-4.642	< 0.001
Left VA pulsatility index in intracranial segment	1.526	0.127	-0.683	0.494	0.513	0.608
Left VA systolic flow velocity in intracranial segment	-4.432	< 0.001	-4.453	< 0.001	-4.227	< 0.001
Left VA diastolic flow velocity in intracranial segment	-5.494	< 0.001	-4.437	< 0.001	-4.686	< 0.001
Mean left VA flow velocity in intracranial segment	-5.256	< 0.001	-4.661	< 0.001	-4.644	< 0.001

*TyG* triglyceride-glucose, *BA* Basilar artery, *ICA* internal carotid artery, *VA* vertebral artery

## Discussion

The disorder that causes the highest rate of disability in middle-aged and older persons worldwide is cerebrovascular disease [23]. Strengthening primary prevention to reduce the incidence of stroke is a crucial strategy for reducing the burden of the disease. The three main risk factors for cerebrovascular diseases are hypertension, diabetes mellitus, and dyslipidemia [2, 24]. A number of diseases, including cerebrovascular disorders, are thought to be at risk due to IR, which is directly related to glucolipid metabolism and body weight [25–27]. Although the clamp test is the gold standard for IR, clinical settings do not typically use it due to its complexity. The TyG index has lately been viewed as a simple and uncomplicated biomarker of IR due to its consideration of lipid factors [28]. We screened the general population for the absence of cerebrovascular disease and divided the study population into two groups based on the TyG index in order to investigate the connection between the TyG index and cervical vascular parameters.

Prior studies have established a substantial correlation between BMI and IR, and high BMI is associated with anomalies in glucolipid metabolism and high blood pressure [29, 30]. Because TyG index reflects IR levels in vivo, participants in the current study with high TyG index had higher blood pressure and Obesity than those with low TyG index. Because TyG index is based on glucose and TG, participants with high TyG index had higher TG, TC, LDL-C, and lower HDL-C, which is consistent with the findings of the preceding study [30]. According to the study, people in the high TyG group tended to be older and to have a higher proportion of men, two elements that also affect the TyG index.

The cerebral basilar artery's hemodynamic characteristics, which reflect the functioning of the cerebral vasculature, are directly accessible through TCD. In this study, we examined the correlation between TyG index and hemodynamic parameters of BA, ICA, and VA to ascertain the effect of TyG index on the vascular function of the neck in the general population. A larger number suggests a higher level of peripheral cerebrovascular resistance, while a lower value indicates a lower level of resistance in the pulsatility index [31]. A risk factor for worsening neurological symptoms in the stroke patients is an increase in BA pulsatility index [32]. In this study, there was no significant difference in BA pulsatility index between the high and low TyG groups, indicating that TyG has no effect on BA vascular resistance, which may be due to the fact that the study population was a general population free of cerebrovascular disease. The fact that right ICA and left VA were higher in the individuals of the high TyG group, while the corresponding contralateral side was not impacted by TyG index, suggests that the increased TyG index appears to have a distinct effect on ICA and VA. The inclusion of a general population in the study and the limited sample size may have contributed to this conclusion. The cerebral artery lumen's size and blood flow are reflected in the blood flow velocity. The relationship between flow velocity and lumen size is inverse when blood flow is constant. Systolic, diastolic, and mean flow velocity in BA, ICA, and VA were lower in the high TyG group than in the low TyG group in the current study. Reduced flow velocity in the BA, ICA, and VA suggest impaired vascular function, though most likely not a blockage of the vascular lumen, but rather harm to the vascular endothelium. Elevated TyG index is strongly associated with atherosclerosis, arterial stiffness, and vascular endothelial cell dysfunction. In the present study, elevated TyG index was found to be associated with reduced BA, ICA, and VA flow velocities in the general population, suggesting the onset of early vascular dysfunction. This may make TyG index a predictor of early cerebrovascular disease in the general population.

Even after controlling for confounding variables, correlation analysis revealed a negative connection between the TyG index and the BA, ICA, and VA flow velocities. Higher blood pressure and issues with glucolipid metabolism were linked to elevated TyG index, both of which affect vascular function. The reduction of vascular endothelial cell function also reduces flow velocity, even if luminal stenosis does not happen in the general population without cerebrovascular illness. However, correlation analysis for the pulsatility index revealed no link between TyG index and carotid vascular pulsatility index, which could be attributed to the absence of overt cerebrovascular illness in the included cohort. Age is a known essential factor impacting vascular function; hence, after controlling for age, there was a minor association between BA and right VA and TyG index; however, the correlation vanished after adjusting for blood pressure. Age-related changes in blood pressure may impact blood flow velocities, which in turn may affect the pulsatility index. This phenomena may also be explained by bias resulting from the study's small sample size.

This study has some limitations. Firstly, as this was a single-center outpatient-based study, it is possible that not all participants had the same results. Secondly, this was a cross-sectional study and therefore could not provide a causal relationship between TyG index and cervical vascular function parameters. Third, factors such as lifestyle may influence outcomes but were not considered in the analysis. And fourth, endothelial function, arterial stiffness, and vascular reactivity indices were not measured, and interventions were not explored in this study. Finally, the TyG index was used to estimate IR levels in this study, and the calculation of a steady-state model assessment index may be needed in the future to validate the results of this study.

## Conclusion

In conclusion, our findings confirm a negative correlation between TyG index with BA, ICA and VA flow velocities, with or without adjustment for confounding factors. This finding shows that elevated TyG index in the general population without cerebrovascular disease appears to be a risk factor for cervical vascular dysfunction.

## Data Availability

Data supporting the results of this study are available upon reasonable request from the first author.
